# Safety Lapses Prior to Initiation of Hemodialysis for Acute Kidney Injury in Hospitalized Patients: A Patient Safety Initiative

**DOI:** 10.3390/jcm7100317

**Published:** 2018-10-01

**Authors:** Adrianna Douvris, Khalid Zeid, Swapnil Hiremath, Pierre Antoine Brown, Manish M. Sood, Rima Abou Arkoub, Gurpreet Malhi, Edward G. Clark

**Affiliations:** 1 Department of Medicine, University of Ottawa, Ottawa, ON K1H 7W9, Canada; adouvris@toh.ca (A.D.); kzeid027@uottawa.ca (K.Z.); rima-n82@hotmail.com (R.A.A.); gmalh035@uottawa.ca (G.M.); 2 Division of Nephrology, Department of Medicine and Kidney Research Centre, Ottawa Hospital Research Institute, University of Ottawa, Ottawa, ON K1H 7W9, Canada; shiremath@toh.ca (S.H.); pibrown@toh.ca (P.A.B.); msood@toh.ca (M.M.S.)

**Keywords:** acute kidney injury, patient safety, hemodialysis

## Abstract

**Background:** Safety lapses in hospitalized patients with acute kidney injury (AKI) may lead to hemodialysis (HD) being required before renal recovery might have otherwise occurred. We sought to identify safety lapses that, if prevented, could reduce the need for unnecessary HD after AKI; **Methods:** We conducted a retrospective observational study that included consecutive patients treated with HD for AKI at a large, tertiary academic center between 1 September 2015 and 31 August 2016. Exposures of interest were pre-specified iatrogenic processes that could contribute to the need for HD after AKI, such as nephrotoxic medication or potassium supplement administration. Other outcomes included time from AKI diagnosis to initial management steps, including Nephrology referral; **Results:** After screening 344 charts, 80 patients were included for full chart review, and 264 were excluded because they required HD within 72 h of admission, were deemed to have progression to end-stage kidney disease (ESKD), or required other renal replacement therapy (RRT) modalities in critical care settings such as continuous renal replacement therapy (CRRT) or sustained low efficiency dialysis (SLED). Multiple safety lapses were identified. Sixteen patients (20%) received an angiotensin converting enzyme inhibitor or angiotensin receptor blocker after AKI onset. Of 35 patients with an eventual diagnosis of pre-renal AKI due to hypovolemia, only 29 (83%) received a fluid bolus within 24 h. For 28 patients with hyperkalemia as an indication for starting HD, six (21%) had received a medication associated with hyperkalemia and 13 (46%) did not have a low potassium diet ordered. Nephrology consultation occurred after a median (IQR) time after AKI onset of 3.0 (1.0–5.7) days; **Conclusions:** Although the majority of patients had multiple indications for the initiation of HD for AKI, we identified many safety lapses related to the diagnosis and management of patients with AKI. We cannot conclude that HD initiation was avoidable, but, improving safety lapses may delay the need for HD initiation, thereby allowing more time for renal recovery. Thus, development of automated processes not only to identify AKI at an early stage but also to guide appropriate AKI management may improve renal recovery rates.

## 1. Introduction

Acute kidney injury (AKI) is a frequent and serious complication of hospitalization, affecting up to 20% of hospitalized patients, and conferring a four-fold increased risk of in-hospital mortality [1]. In-hospital mortality increases with increasing severity of AKI, with the highest mortality observed in patients that require renal replacement therapy (RRT) [2,3]. Systematic reviews have shown that AKI is associated with long term consequences including increased mortality, chronic kidney disease (CKD), and progression to end-stage kidney disease (ESKD) [4,5]. Although the use of RRT for AKI is life-sustaining when urgently indicated, it is costly [6,7] and may be harmful for renal recovery [8].

Currently, there are no effective pharmacological interventions for AKI, [9] and management is aimed at limiting further kidney injury and reducing the likelihood that acute indications for RRT will develop prior to renal recovery [8]. The progression of AKI may be limited by timely diagnostic workup if worsening renal injury (and the consequent need for RRT) is prevented by limiting the use of nephrotoxic medications [10], and iodinated contrast dye [11], although there is debate in the literature surrounding the association between intravenous iodinated contrast and AKI in hospitalized patients [12]. Limiting excess dietary or intravenous potassium may increase the likelihood of recovery prior to hyperkalemia becoming an indication for RRT, however there is a paucity of data in this area. Nonetheless, there is some evidence that safety lapses in the care of hospitalized patients with AKI are frequent [13].

Early identification of AKI in hospitalized patients using electronic alerts has the potential to reduce the likelihood of AKI progression and need for RRT [14,15,16], however, this has not been supported by the literature up to now. A large single-center randomized controlled trial assessing automated electronic clinician notifications did not reduce death or need for RRT [17]. In addition, a recent systematic review of six studies of electronic alerts for AKI found no improvement in survival or need for RRT, with variable impact on processes of care [18]. The lack of efficacy of these early alerts may relate to AKI being a syndrome of many causes that require different interventions. Another issue is that alerts may not trigger significant changes in care processes, such as medication review with cessation of nephrotoxic medications, or IV fluid administration [19]. Consequently, to improve outcomes and reduce the need for unnecessary RRT, it may be first necessary to identify the processes that are most likely to lead to iatrogenic harm.

As such, we undertook a study to characterize safety lapses that might have contributed to the need for potentially avoidable hemodialysis (HD) for AKI patients at our center. Given that patients who initiate forms of RRT for AKI other than HD (e.g., continuous renal replacement therapy (CRRT) or slow low-efficiency dialysis (SLED)/prolonged intermittent RRT (PIRRT)) typically do so in the intensive care unit (ICU) setting due to hemodynamic instability, we sought to focus on more stable patients initiating HD for AKI with respect to their preceding exposure to nephrotoxic oral medications and incorrect dietary orders (while still including patients if their HD for AKI was ultimately initiated in the ICU setting).

## 2. Experimental Section

### 2.1. Study Design and Setting

We conducted a retrospective chart review of patients who started treatment with HD for AKI while hospitalized at The Ottawa Hospital (TOH) between 1 September 2015 and 31 August 2016. TOH is a tertiary care academic medical center with 1061 inpatient beds that services a population of approximately 1.2 million people across Eastern Ontario, Canada [20]. TOH has over 50,000 patient admissions annually at three campuses (Ottawa General Hospital; Ottawa Civic Hospital and University of Ottawa Heart Institute) [20]. At the time of this study, TOH did not have computer physician order entry (CPOE) for medications or investigations other than imaging studies.

Prior to the start of the study, approval for waived patient consent was obtained from TOH research ethics board.

### 2.2. Patient Population, Inclusion and Exclusion Criteria

Patients were identified retrospectively for screening using consecutive nephrology billing codes that had been submitted for new, inpatient hemodialysis starts.

Inclusion criteria were: hospitalized patients; aged 18 years or older; with AKI (as defined below); who required initiation of RRT in the form of intermittent HD. We excluded patients who: required HD within 72 h of admission (as such cases were considered to be more likely reflective of severe AKI at the outset of hospitalization in which RRT was less likely to be avoidable); patients with ESKD; RRT started for a reason other than AKI (e.g., intoxication, hypothermia); or if RRT was started using a modality other than HD (i.e., CRRT or SLED). For patients re-admitted to hospital requiring HD on re-admission, we gathered data from both admissions to capture the initial AKI that did not resolve.

### 2.3. Data Sources and Data Collection

Data was collected through a retrospective chart review of electronic medical records. Electronic medical records included relevant investigations (labs and imaging), as well as consultation notes, scanned progress notes, physician orders and medication administration records.

Two investigators (AD, KZ) independently screened charts then reviewed the electronic charts of included patients and collected data on their baseline demographics, co-morbidities and iatrogenic processes. All charts were reviewed by both investigators and disagreements between the two primary chart reviewers on aspects of data collection were resolved by a third investigator (E.C.) for consistency of data collection.

Data was extracted from the inpatient chart, and recorded on data collection forms before being entered into an Excel database. The onset of AKI was determined as the first instance that patients fulfilled the serum creatinine (SCr)-based Kidney Disease Improving Global Outcomes (KDIGO) criteria for AKI, which corresponds to a rise in SCr of ≥1.5 times baseline over 7 days or an increase in SCr by at least 26.5 μmol/L [21] within 48 h. Baseline SCr was calculated using the lowest available outpatient SCr within 12 months [22]. When none was available, the first SCr following hospitalization was used [22].

### 2.4. Outcomes and Analysis

Our outcomes of interest were the frequency with which specific iatrogenic processes may have contributed to the need for RRT in our population (described below). Other outcomes included time from AKI diagnosis to initial management steps, including Nephrology referral (which occurred at some point for all included patients as it is a necessary pre-requisite to receiving HD at our institution).

To identify delays in AKI identification and initial management, the timing of Nephrology consultation and HD initiation relative to the onset of AKI was reported as the median number of days with interquartile ranges. The possible causes of AKI were determined from admission notes, Nephrology consultations, and progress notes. Renal investigations post-AKI, including urine studies and imaging, were recorded. IV fluid administration within 24 h of AKI onset when AKI was recorded to be ‘pre-renal’ from hypovolemia, was recorded. We did not differentiate between a bolus or infusion of IV crystalloid as ordering practices varied between prescribing physicians depending on clinical context. The number and type of iodinated contrast imaging studies after AKI were recorded. The indications for first HD were obtained from Nephrology consult and progress notes.

Iatrogenic events and processes relating to AKI and hyperkalemia were recorded. This included the administration of certain medications after the onset of AKI including non-steroidal anti-inflammatory drugs (NSAIDs), angiotensin converting enzyme inhibitors (ACEi), angiotensin receptor antagonists (ARBs) and potassium-sparing diuretics or aldosterone inhibitors. The administration of oral K^+^ supplements, and IV solutions containing at least 10 mmol/L of potassium after the onset of AKI and when serum potassium was ≥5.0 mmol/L was also recorded. Ordered diets were recorded, including failure to order ‘renal’ or low potassium diet after onset of AKI who were ultimately dialyzed with hyperkalemia (defined as potassium ≥ 5.5 mmol/L). The frequency of iatrogenic events was calculated.

The collected data was also analyzed qualitatively and selected cases that were felt by the investigators to be representative of particular patient safety lapses in this population are reported in a narrative synthesis.

## 3. Results

### 3.1. Patient Demographics and AKI Information

**Figure 1:**
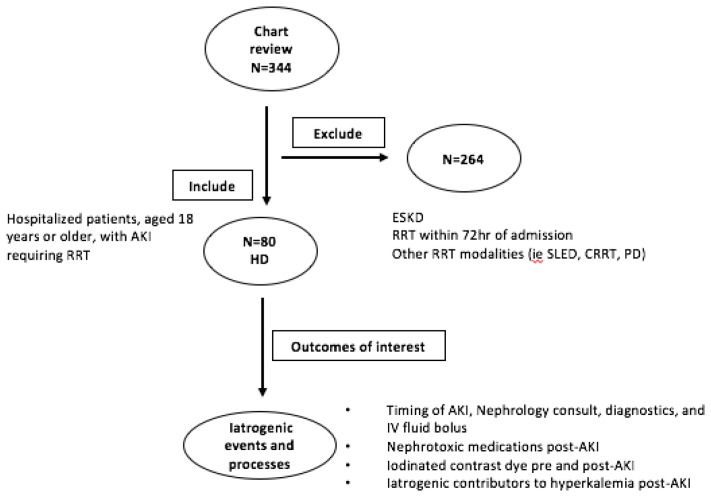
Summary of study design for this retrospective chart review. A total of 344 electronic inpatient records were reviewed, and 80 consecutive hospitalized patients meeting inclusion criteria were included. Data was collected for qualitative assessment of iatrogenic events and processes that may have contributed to the need for RRT (in the form of HD) for AKI. AKI, acute kidney injury; RRT, renal replacement therapy; HD, intermittent hemodialysis; ESKD, end-stage kidney disease; SLED, slow low efficiency dialysis; PD, peritoneal dialysis.

We reviewed 344 charts and excluded 264 patients for a total of 80 consecutive patients, over a one-year period, treated with at least one HD session for AKI while hospitalized. The process is outlined in Figure 1.

Table 1 summarizes baseline patient characteristics. The mean age of patients was 65 years old and over half were documented to have CKD. The average baseline serum creatinine (SCr) was 1.9 mg/dL (138 μmol/L). All patients were initiated on HD for AKI at the Ottawa Hospital (TOH), but 19 patients (23.7%) were initially admitted to other hospitals and transferred to TOH for specialty or intensive care. In-hospital mortality was 26% (21 patients) and the median length of stay in hospital was 28.0 days [IQR 16.3–53.5]. Overall, 64 patients (80%) were initially admitted to a medical service and 16 (20%) to a surgical service. Thirty patients (38%) required critical care (in an intensive care unit (ICU), cardiac care unit, or cardiac surgery ICU) by the time of HD initiation. The most common admission diagnosis was sepsis (in 25 patients (31%)) but cardiac causes were listed for 27 patients (34%) (classified as acute coronary syndrome in 13 patients (16%) and CHF in 14 patients (18%) overall).

Supplementary Figure S1 details the etiology of AKI for included patients, as determined by documentation in each patient’s chart from admitting services and Nephrology consultants. More than one etiology was implicated in 51 patients (64%).

Timing of AKI recognition, work-up, and management is reported in Table 2. As summarized in Table 2, half of our patients met criteria for AKI at the time of admission. Of those who developed AKI in hospital, the median time to AKI was 4.5 days. The time from AKI to Nephrology consultation and HD initiation was 3 days and 6 days, respectively. With respect to diagnostic work up for AKI, urinalysis with microscopy and urine electrolytes were assessed for 61 patients (76%) and 45 patients (56%), respectively. The median time between AKI and obtaining urine electrolytes was 3 days. Fifty-three (66%) patients underwent renal ultrasonography or another form of abdominal imaging that could rule out hydronephrosis. Lastly, of the 35 patients with pre-renal AKI secondary to hypovolemia, 29 (83%) received an IV fluid administration of crystalloid or colloid within 24 h of AKI onset.

### 3.2. Nephrotoxins, Medications, Hyperkalemia and Indications for Dialysis

Table 3 summarizes the frequency of selected medications and exposure to contrast dye after the onset of AKI and prior to HD. Either an ACEi or ARB was given post-AKI in 16 patients (20%) and 11 patients (14%) were given spironolactone. Three patients (4%) received both ACEi or ARB plus spironolactone after AKI. One patient (1%) received NSAIDs post-AKI. In the post-AKI period, 15 patients (19%) and 9 patients (11%) received either intravenous or intra-arterial contrast, respectively.

**Figure 2:**
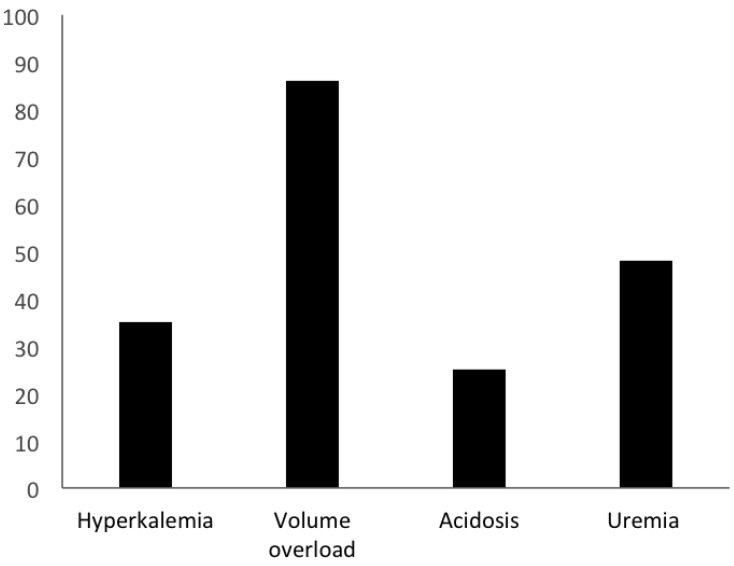
Indications for initiation of hemodialysis (*n* = 80). Legend: Percentage of patients with a particular indication for initiation of hemodialysis. Fifty-one patients (64%) had two or more indications present.

Figure 2 illustrates the frequency of the presence of indications for initiation of HD at the time it was started. Volume overload was the most common indication, present in 69 patients (86%). Uremia was cited as an indication in 40 patients (50%). Hyperkalemia (with a serum potassium ≥ 5.5 mmol/L in all such cases) was documented as an indication for HD in 28 patients (35%). Most patients had multiple indications for HD initiation, and hyperkalemia was only an isolated indication for 2 patients (3%). For the 28 patients dialyzed with hyperkalemia as an indication for initiation of HD, all had serum potassium ≥ 5.5 mmol/L and 6 (21%) had serum potassium ≥ 6.0 mmol/L at HD initiation.

Table 4 highlights iatrogenic contributors to hyperkalemia. Of the 28 patients dialyzed with hyperkalemia, 13 (46%) were not given a low potassium diet and 3 (11%) were receiving either an ACEi, ARB or spironolactone; one (4%) received potassium supplements in addition to spironolactone. Further, of the 6 patients with serum potassium ≥ 6.0 mmol/L at HD initiation, 3 were not provided with low potassium diets, and 2 received potassium supplements or potassium sparing diuretics.

### 3.3. Summary of Representative Cases

Table 5 summarizes four cases that highlight safety lapses that may have contributed to the need to initiate HD after the onset of AKI.

## 4. Discussion

Our study of 80 consecutive inpatients who required hemodialysis for AKI after at least 72 h of hospitalization revealed that safety lapses occur frequently and may have contributed to the need for initiation of HD in some instances. This is consistent with previous studies that have demonstrated safety lapses occur frequently in patients who die in hospital with a primary admission diagnosis of AKI [13] and in end-stage kidney disease patients admitted to surgical services [23].

Our results suggest deficiencies in diagnostic testing to determine the etiology of acute kidney injury. In particular, it was notable that only 61 patients (76%) had urinalysis testing after AKI while the KDIGO Clinical Practice Guidelines for AKI [21] suggest that urinalysis testing is necessary to ensure a complete diagnostic work-up for AKI. Another safety lapse we discovered was the frequent failure to order low potassium diets in patients with AKI who ultimately started dialysis with elevated serum potassium levels. Low potassium diets were ordered in less than half of such patients. Although there is no published data in the literature on low potassium diets and RRT initiation in patients with AKI, we feel this is a low risk intervention that has the potential to delay HD in the AKI population. As well, supplemental potassium or medications known to increase the serum potassium level were continued in many such patients. This suggests that our institution might improve care through an automated trigger to review these particular medications after the onset of AKI and/or elevated serum potassium. For potassium supplements, automatic substitution to a *prn* order restricted according to serum potassium values could also be useful.

Another issue that our study identified is that Nephrology consultation was often delayed, with a median time from AKI to consultation of three days. Studies of hospitalized patients with AKI, including a recent systematic review and meta-analysis [24], have shown that delayed Nephrology consultation for AKI is associated with increased in-hospital mortality in both non-critically ill [25] and critically ill patients [24,26,27], increased risk of requiring RRT [25], and increased dialysis dependence rates upon hospital discharge [26]. One particular study found that for hospital-acquired AKI (using the same KDIGO definition [21] as our study), nephrology assessment within 18 h was associated with significantly fewer patients progressing to a 2.5-fold increase in SCr level from admission [28]. We also found that, for patients who had urine electrolyte testing performed, it was done a median of three days after the AKI onset. Although the clinical utility of urine electrolyte testing for AKI is itself debatable, it does suggest a substantial time lapse between the onset of AKI and investigations related to AKI and that AKI may be under-recognized. Further evidence that AKI is under-recognized is that only 29 of the 35 patients (83%) with a pre-renal element to their AKI received IV fluids as a bolus or infusion within 24 h of AKI. Overall, our findings suggest that an automated trigger for nephrology assessment (and initial diagnostic testing, including serial SCr measurements, urinalysis, microscopy, ultrasound, and initial management strategies including medication review and volume status) might be one avenue to reducing the likelihood of AKI patients progressing to require RRT initiation at our institution. We recognize that this could add substantial burden to the existing inpatient Nephrology service and may require a dedicated team to address assessments.

The main strength of our study is that it involved a comprehensive case-by-case review to capture clearly defined, pre-specified, safety lapses. However, there are many important limitations. The study was not comprehensive and did not evaluate a myriad of other possible medication or treatment-related safety lapses that could have a bearing on AKI progression to HD initiation. As well, our study was not able to determine the clinical significance of any safety lapses with respect to whether they impacted the subsequent requirement for HD as we did not assess a comparator group of AKI patients who did not progress to require HD. Furthermore, many ‘iatrogenic’ processes are likely unavoidable. For example, although it was not possible to quantify, on the basis of our case-by-case analysis, the vast majority of contrast imaging was clearly indicated in the overall context of patients’ clinical management despite its potential nephrotoxicity. As briefly discussed earlier, there is also controversy in the literature regarding the association between IV iodinated contrast dye and AKI [12]. A final limitation relates to generalizability: some of the lapses in safety might be less likely to occur in institutions utilizing CPOE. Furthermore, our experience might not be generalizable to the community hospital setting where specialist consultations or subspecialty admitting services are less likely to be available.

Despite its limitations, this study clearly highlights several care processes to target for improvement. The development of an automated trigger to ensure discontinuation of medications that are either nephrotoxic and/or promote hyperkalemia could be beneficial. As well, an automated review of diet orders (to ensure a low potassium diet, when indicated) and automated triggers for nephrology referral soon after AKI onset could increase the frequency with which renal recovery occurs prior to hemodialysis being required.

## Figures and Tables

**Table 1:** Baseline patient characteristics (*n* = 80).

Mean age in years (SD)	65.5 (+/− 15.4)
Male sex, *n* (%)	50 (62)
Mean baseline serum creatinine in mg/dL (SD)	1.6 (+/− 0.9)
Co-morbidities, *n* (%)	
Hypertension	54 (68)
Diabetes mellitus	47 (59)
Chronic kidney disease	43 (54)
Congestive heart failure	33 (41)
Peripheral vascular disease	13 (16)
Home medications, *n* (%)	
Thiazide diuretic or furosemide	(54)
ACEi or ARB	(50)
Metformin	(23)
Spironolactone	(15)
Admission diagnoses *	
Sepsis	26 (33)
Congestive heart failure	17 (21)
Acute coronary syndrome	14 (18)
Acute kidney injury	15 (19)
Malignancy	8 (10)
Hospitalization and outcomes	
Admitted upon hospital transfer, *n* (%)	(23.7)
Median hospital length of stay, days (IQR)	28.0 (16.3–53.5)
In-hospital mortality, *n* (%)	(26.2)

* Patients could have more than one diagnosis recorded as the reason for admission. SD, standard deviation; IQR, interquartile range; ACEi, angiotensin converting enzyme inhibitor; ARB, angiotensin receptor blocker

**Table 2:** Diagnosis and management of Acute Kidney Injury, *n* = 80 *.

AKI present at admission, *n* (%)	40 (50.0)
Median time from admission to AKI, days (IQR)	4.5 (2.0–11.2)
Median time from AKI to Nephrology consult, days (IQR)	3.0 (1.0–5.7)
Median time from AKI to first hemodialysis, days (IQR)	6.0 (4.0–11.0)
Tests and initial management, *n* (%)	
IV fluid administration within 24 h for pre-renal AKI, *n* = 35	29 (83)
Urinalysis and routine microscopy	61 (76)
Renal ultrasound	53 (66)
Urine electrolytes	45 (56)

* Unless otherwise specified. AKI, acute kidney injury; IQR, interquartile range

**Table 3:** Selected iatrogenic medications and contrast exposure after Acute Kidney Injury.

Medications, *n* (%)	
ACEi or ARB	16 (20)
Spironolactone	11 (14)
NSAIDs	1 (1)
Aminoglycoside antibiotic	1 (1)
Contrast exposure, *n* (%)	24 (30)
Intravenous	15 (19)
Intra-arterial	9 (11)

ACEi, angiotensin converting enzyme inhibitor; ARB, angiotensin receptor blocker; NSAIDs, nonsteroidal anti-inflammatory drug.

**Table 4:** Iatrogenic contributors to hyperkalemia after Acute Kidney Injury.

Occurrence of hyperkalemia (*n* = 80), *n* (%)	
During admission, after AKI	33 (41)
As an indication for dialysis	28 (35)
Safety lapses in patients with hyperkalemia as a subsequent indication for hemodialysis (*n* = 28), *n* (%)	
Low potassium diet not ordered	13 (46)
Oral potassium supplements given while serum potassium ≥ 5.0 mmol/L	2 (7)
ACEi, ARB and/or spironolactone given while serum potassium ≥ 5.0 mmol/L	6 (21)

AKI, acute kidney injury; ACEi, angiotensin converting enzyme inhibitor; ARB, angiotensin receptor blocker.

**Table 5:** Selected cases that highlight safety lapses in patients requiring hemodialysis after Acute Kidney Injury.

Admission Diagnoses	Indication(s) for HD	Summary of Events after AKI and Prior to Initiation of HD
Lymphoma, AKI	Hyperkalemia, Volume overload	Diuresis then IV contrast for CT scan; worsening AKI Spironolactone and potassium supplements continued despite serum potassium 5.5 mmol/L.
Sepsis, NSTEMI and AKI	Volume overload	Long-acting CCB, BB and nitropatch continued despite relative hypotension; CT with IV contrast Given 9 L of IV crystalloid for refractory hypotension while oligoanuric with subsequent development of pulmonary edema.
NSTEMI, then AKI *	Volume overload Hyperkalemia	CKD with baseline Cr 200 Discharged 24 h after coronary angiogram with Cr 210, K 5.6. Was continued on ARB and started on NSAID at discharge. Re-admitted 48 h later with oliguric AKI, serum potassium up to 6.3 mmol/L, volume overload.
Anemia, AKI	Respiratory failure	Late Nephrology referral (9 days post-admission with AKI non-responsive to IV fluids Urinalysis at admission showed microscopic hematuria, proteinuria with hypoalbuminemia. GN work up initiated by Nephrology, including renal biopsy. Transfer to ICU for respiratory failure; initiated HD, and started plasmapheresis, cyclophosphamide, steroids for microscopic polyangiitis.

AKI, acute kidney injury; CT, computed tomography; IV, intravenous; CCB, Calcium channel blocker; BB, beta-blocker; ARB, angiotensin receptor blocker; NSAID, non-steroidal anti-inflammatory drug; GN, glomerulonephritis; HD, intermittent hemodialysis. * This case was excluded from our study cohort because this patient was initiated on hemodialysis within 48 h of admission. It has been included in this table to highlight a patient safety issue around this patient’s discharge post-angiogram that was still detected on chart review.

## Supplementary Materials

The following are available online at http://www.mdpi.com/2077-0383/7/10/317/s1. Supplemental Figure S1: Etiology of Acute Kidney Injury (*n* = 80).
