# Supplementary material for: Safety Lapses Prior to Initiation of Hemodialysis for Acute Kidney Injury in Hospitalized Patients: A Patient Safety Initiative
Source: J Clin Med. 2018 Oct 1;7(10):317. doi: 10.3390/jcm7100317 (PMC6211106; doi:10.3390/jcm7100317)
Supplement: Supplementary file 1 [file jcm-07-00317-s001.pdf]

Supplemental Figure S1

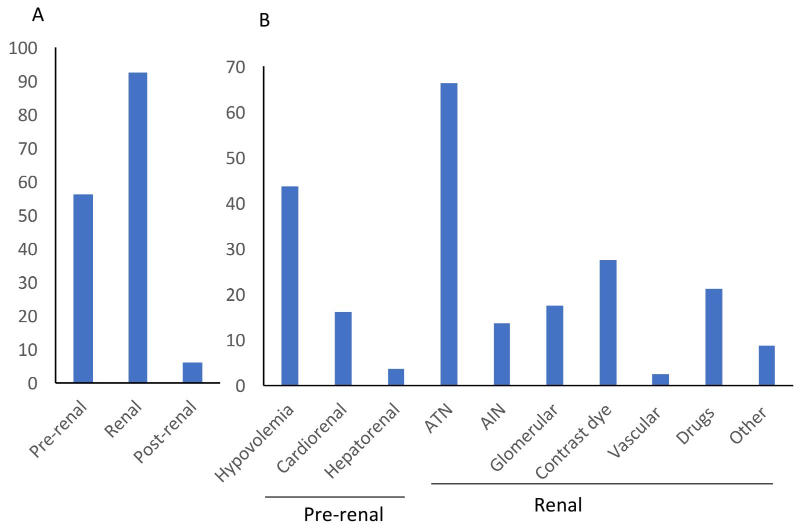

S1. Presumed AKI cause(s) represented as percentage within the study population. **(A)** Proportion of AKI within each major category. **(B)** Percent of pre-renal and renal etiologies of AKI. Pre-renal AKI includes mechanisms of reduced effective circulating volume. Renal AKI includes glomerular, tubulointerstitial, and vascular causes. It also includes nephrotoxins. Other causes of renal AKI (8.8%) include malignant mechanisms (tumour lysis,myeloma kidney), and thrombotic microangiopathy (TMA). The causes of AKI were gathered from admission note, Nephrology consult, progress notes, or a combination of these. There was more than one AKI cause provided for each patient.
